# Investigating the Impact of Extruded Dehulled Adlay with Specific In Vitro Digestion Properties on Blood Lipids in Subjects with Mild to Moderate Dyslipidemia

**DOI:** 10.3390/foods11040493

**Published:** 2022-02-09

**Authors:** Chieh Chung, Ting-Yu Chao, Hong-Jhang Chen, Gui-Ru Xie, Wenchang Chiang, Shu-Chen Hsieh

**Affiliations:** 1 Institute of Food Science and Technology, National Taiwan University, Taipei City 106, Taiwan; totoya7627@gmail.com (C.C.); d03641001@ntu.edu.tw (T.-Y.C.); fsthjchen@ntu.edu.tw (H.-J.C.); verahsieh1212@gmail.com (G.-R.X.); chiang@ntu.edu.tw (W.C.); 2 Department of Dietetics and Nutrition, Heping Fuyou Branch, Taipei City Hospital, Taipei City 100, Taiwan

**Keywords:** dehulled adlay, dietary intervention, dyslipidemia, slowly digestible starch

## Abstract

Dyslipidemia, a major risk factor for cardiovascular diseases (CVDs), is modifiable by diet and lifestyle changes. A large population with mild to moderate dyslipidemia is at risk of developing CVDs, and early initiation of preventive measures can avert advancing into severe medical conditions. Studies suggest increasing slowly digestible starch (SDS) in diets can help lower blood lipids. We processed dehulled adlay, a cereal rich in bioactive compounds, such as polyphenols and phytosterols, into an instant meal by extrusion and milling and then assessed its starch composition and in vitro digestibility. The dehulled adlay was found to consist of 32% SDS and resistant starch combined. Then, eligible subjects with dyslipidemia were recruited to explore the adlay’s hypolipidemic potential, safety, and acceptability. Subjects consumed the dehulled adlay as the sole carbohydrate source in their breakfast, without changing other components in the diet or lifestyle, for 12 weeks. After intervention, serum total cholesterol (TC) decreased significantly in subjects with hypercholesterolemia. In addition, both TC and triglyceride levels decreased significantly in those above 50 years old. In conclusion, the extruded dehulled adlay displays potential for favorably modulating blood lipids, and the effect is more pronounced in the middle-aged population.

## 1. Introduction

Cardiovascular diseases (CVDs) rank as the first leading cause of death globally [1]. According to the Global Burden of Disease Study 2019 [2], the prevalence of CVDs nearly doubled in the past 20 years, and 523 million people worldwide are currently affected. Several modifiable risk factors contribute to cardiovascular health, including body mass index, smoking, and health conditions, such as elevated blood sugar and blood lipid levels [3]. Dyslipidemia is a term that describes unhealthy levels of lipids in the blood, and it is crucially associated with nutritional overload and physical inactivity [4]. Physiological lipid metabolism can be divided into three parts: exogenous (dietary), endogenous (hepatic lipogenesis), and reverse cholesterol transport [5]. After dietary fats are digested, cholesterol (TC) and triglycerides (TG) are assembled into chylomicrons that are absorbed by the intestinal villi, enter the circulation, and are distributed to adipose tissues for storage or acted upon by lipoprotein lipase (LPL) into glycerol and fatty acids that may be used for fuel. Endogenously, the liver synthesizes very-low-density lipoprotein (VLDL), which is cleaved by LPL into intermediate-density lipoprotein (IDL) and further into low-density lipoprotein (LDL) to release free fatty acids. High-density lipoprotein (HDL) functions to transport excessive cholesterol from peripheral tissues back to the liver. 

Exposure to even mildly elevated cholesterol levels increases the risk of developing coronary heart disease later in life [3]. Early measures, such as lifestyle changes, can be taken to prevent the development of severe medical complications. Diet modifications to incorporate high amounts of fruits and vegetables, nuts and legumes, whole grains, and moderate amounts of dairy and seafood can be adopted to prevent CVDs [6]. Such dietary components have a lower glycemic index (GI) than refined, highly processed foods, meaning they impose a relatively low effect on blood glucose variations after consumption. Food starches are the primary determinant of glycemic response; based on their rate of digestion, starches can be classified into three types: rapidly digestible starch (RDS), slowly digestible starch (SDS), and resistant starch (RS), in decreasing order of glycemic stimulation. Several studies have reported the beneficial effect of SDS in managing diabetes, heart disease, and obesity [7,8].

Adlay (*Coix lachryma-jobi* L.), also called Job’s tears, is a cereal cultivated as a food and medicinal crop in Asia [9]. Adlay seeds are rich in functional compounds, such as dietary fibers, polyphenols, as well as phytosterols. Various bioactivities have been reported for adlay, including anti-oxidation, anti-allergy, hypolipidemia, and immunoregulation, proven in both in vitro and in vivo studies [10]. Our team previously developed a dehulled adlay snack with a higher SDS and RS content by extrusion processing as an alternative snacking option for controlling glycemic response. In order to improve the hard texture of the snack to fit the demand of the elderly, we modified the form by pulverizing, which would inevitably influence the original composition of starches. There are two aims of this study, therefore. First, to determine the starch composition of the dehulled adlay powder. Second, to investigate the effects of the dehulled adlay powder on subjects with mild to moderate dyslipidemia in a dietary intervention study.

## 2. Materials and Methods

Dehulled adlay of cultivar Taichung No. 4 (TCS4) was purchased from Taichung District Agricultural Research and Extension Station, Council of Agriculture, Taiwan. The white bread (Uni-President Inc., Tainan, Taiwan) used as the reference sample in the in vitro digestibility experiments was purchased from a local convenience store. D-glucose assay kit (K-GLUC) was from Megazyme International, Ltd. Other chemicals were from Sigma-Aldrich, Merck, or J. T. Baker.

### 2.1. Sample Preparation

Dehulled adlay was prepared by extruding with the following conditions: feed moisture at 22–25%, screw speed at 130–267 rpm, and a temperature gradient of 100–150 °C. The extruded dehulled adlay (hereafter referred to as EDA) was cooled, dried, milled to fine powder with a high-speed pulverizer (Rong Tsong Precision Technology Co., Taichung City, Taiwan), and then passed through a 100-mesh sieve. The filtrate was collected and used as the samples for subsequent experiments. The reference sample was prepared from white bread with the edges removed and the center cut into 5 × 5-cm squares.

### 2.2. Solutions Preparation

Pepsin solution was prepared by adding 0.33 g pepsin and 0.33 g guar gum to 67 mL of 0.05 M HCl and stirring with a magnetic bar for 15 min until totally dissolved. Pancreatin/amyloglucosidase mixture was prepared by dissolving 6 g pancreatin in 40 mL distilled water and centrifuging at 1500× *g* for 10 min. Then, 30 mL of the supernatant was combined with 1.33 mL amyloglucosidase solution (140 amyloglucosidase unit/mL) followed by the addition of 2 mL distilled water. The enzyme solutions were prepared just before use.

### 2.3. In Vitro Starch Digestibility Assay

This method is based on Englsyt [11] and Goñi [12] and modified to comply with the glycemic response to foods in Asian people based on previous work from our lab (unpublished) and described as follows. EDA powder was weighed to 1 g in a flask, followed by the addition of 10 mL of pepsin solution, and then incubated at 37 °C for 30 min in a shaking water bath at 130 rpm to simulate stomach digestion. After 30 min, 10 mL of 0.1 M sodium acetate buffer (pH 5.2) in saturated benzoic acid and three 15 mm-diameter glass balls were added to the solution. A sample of 100 μL was transferred to a tube containing 1.8 mL distilled water, which was considered to be at 0 min. Then, 5 mL of pancreatin/amyloglucosidase mixture solution was added to each sample to simulate intestinal digestion, and 100 μL of solution was sampled at 10, 20, 30, 45, 60, 120, and 180 min separately following enzyme addition to a tube containing 1.8 mL of distilled water; then, the solution immediately placed in 100 °C dry bath for 20 min to inactivate enzyme reaction. Afterward, each sampling tube was centrifuged at 1500 × g for 10 min, and 30 μL of supernatant was then transferred to 1 mL of glucose oxidase/peroxidase reagent from the K-GLUC kit and incubated at 50 °C for 20 min. Triplicates of 200 μL of each sampling time for each sample were transferred to a 96-well plate along with standard glucose solutions at 0, 0.125, 0.25, 0.5, 0.75, and 1 mg/mL. Absorbance was measured at 510 nm using a microplate reader (Epoch™ Microplate Spectrophotometer, BioTek Instruments, Winooski, VT, USA) to determine the glucose content by interpolation using the standard curve.

The starch hydrolysis rate for each sampling time was calculated by Equation (1):

(1) $$\mathrm{Starch}\;\mathrm{hydrolysis}\;\mathrm{rate}\;\left(\%\right)=\frac{\mathrm{glucose}\left(\frac{\mathrm{mg}}{\mathrm{mL}}\right)\times 0.9\times 19\times 25}{\mathrm{total}\;\mathrm{starch}\;\mathrm{in}\;\mathrm{sample}\;\left(\mathrm{mg}\right)}\times 100$$

where 0.9 is the conversion factor (162/180) from starch (empirical MW: 162) to glucose (MW: 180), 19 is the dilution factor (100 μL sample added to 1.8 mL of distilled water), and 25 is the total reaction volume in mL.

The kinetics of enzymatic starch hydrolysis for each sample follows a first order equation, from which we can derive the area under the curve (AUC) of the sample [12]:

(2) $$C_{t}=C_{\infty }\left(1-e^{-kt}\right)$$

(3) $$\mathrm{AUC}=C_{\infty }\left(t_{f}-t_{0}\right)-\left(C_{\infty }/k\right)\left[1-exp-k\left(t_{f}-t_{0}\right)\right]$$

where C_t_ is hydrolysis rate (%) at each sampling time *t*, C_∞_ is the starch hydrolysis rate (%) at equilibrium at the end of the 180 min reaction, k is the kinetic constant, *t_f_* is the final time (180 min), and *t*_0_ is the initial time (0 min). 

The percentages of different starch fractions, namely rapidly digestible starch (RDS), slowly digestible starch (SDS), and resistant starch (RS), were calculated by the following equations according to Englsyt [11]:

(4) $$\mathrm{RDS}\;\left(\%\right)=\left(G_{20}-G_{0}\right)\times 0.9$$

(5) $$\mathrm{SDS}\;\left(\%\right)=\left(G_{120}-G_{20}\right)\times 0.9$$

(6) $$\mathrm{RS}\;\left(\%\right)=100-RDS-SDS$$

where *G*_0_, *G*_20_, and *G*_120_ are glucose content (%) released at 0, 20, and 120 min following pancreatin/amyloglucosidase digestion. 

### 2.4. Phytosterols and Phenolic Compounds Analysis

The analysis of phytosterols was performed according to Sorensen and Sullivan [13] with some modifications. GC–MS analyses were performed on an Agilent 5975 mass spectrometer (electron impact ionization, 70 electron volts; Agilent, Santa Clara, CA, USA) connected to a 7890 gas-chromatography fitted with a fused silica capillary column (HP-5MS, 0.32 mm × 25 m, 0.17-μm film thickness; Agilent). The oven temperature was maintained at 190 °C for 2 min, elevated to 230 °C at a rate of 20 °C/min, and then maintained at 250 °C for 40 min. Helium was used as the carrier gas at a flow rate of 2.0 mL/min. The injection volume was 1 μL. Temperatures of ion source and transfer line were set at 200 °C and 300 °C, respectively. Argon was used as the collision-induced dissociation gas at a pressure of 1.5 mTorr. The standard curves of campesterol, stigmasterol, and β-sitosterol (Sigma-Aldrich, Saint Louis, MO, USA, 0.1–20 µg/L with the correlation coefficient (r) above 0.9992 in all cases) were performed for quantification. The limit of quantification (LOQ) was 0.1 μg/mL, calculated at a signal-to-noise ratio (S/N) of 10. The standard curves for each of the three phytosterols (Figures S1–S3) are provided in Supplementary Materials.

Phenolic compounds in the EDA powder were identified and quantified according to the method described by Tanriseven et al. [14] using LC-ESI-MS/MS. Samples were subjected to a 0.22-μm nylon membrane filter prior to injection. A UHPLC system (Ultimate 3000, Thermo Fisher Scientific, Bremen, Germany) is equipped with a quadrupole orbital trap mass spectrometer (Q-Exactive, Thermo Fisher Scientific, Waltham, MA, USA) with an electrospray ionization (ESI) interface. Kinetex^®^ 1.7 μm Phenyl-Hexy 100 Å, LC Column (150 × 2.1 mm) (Torrance, CA, USA) was used in the analyses. The elution was carried out using double-distilled water containing 0.1% formic acid (eluent A) and acetonitrile containing 0.1% formic acid (eluent B). The flow rate was 400 μL/min with the following gradient: 0 min 5% B; 0–50 min 55% B; 50–55 min 80% B; and 55–65 min 100% B. The temperature of autosampler was set at 40 °C. The injection volume was 5 μL. The positive model was performed, and the scan range was from *m/z* 100~1000. Data acquisition was done by the data-dependent MS2 (ddMS2) mode. In the absence of commercial standards, the ten phenolic compounds listed were identified according to the accurate mass (MS1), isotopic pattern, and product fragment ions (MS2) using Compound Discoverer 3.0 (Thermo Fisher Scientific, Waltham, MA, USA). The mass tolerance is 5 ppm. Semi-quantitation of the phenolic compounds was done using the standard curve (0.04–0.66 μg/mL) of a structurally related substance, quercetin (Sigma-Aldrich, Saint Louis, MO, USA) and expressed in μg/mL of quercetin equivalents (QE). The correlation coefficient (r) of the quercetin standard curve was 0.9982; the LOQ was 0.04 μg/mL, calculated at an S/N of 10.

### 2.5. Human Study Design

This study was performed following the Assessment Guidelines of Health Foods for the Regulation of Blood Lipids announced by the Ministry of Health and Welfare, Taiwan (Amendment No. 0960403114). This study was a prospective, open label, interventional study using a single-group pretest-posttest design, with a three-month dietary intervention period. The main purpose of this study was to offer insight into the hypolipidemic potential of the EDA in addition to its safety and acceptability within the target population. Volunteers were recruited through posters in public bulletin boards in communities, universities, and hospitals. Randomization and blinding to treatment were not applicable due to the single-arm design of this study, and investigators were aware of possible biases, such as placebo effect, overestimation of efficacy, and in the reporting of adverse events. Detection bias was minimized by outsourcing the measurement of blood lipid levels to an external contracted clinical center.

In this study, we aimed to target dyslipidemia in terms of hypertriglyceridemia, hypercholesterolemia, high low-density lipoprotein cholesterol (high LDL), and low high-density lipoprotein cholesterol (low HDL). Inclusion criteria included 30-80 years of age and having one or more fasting serum lipids above optimal levels and/or on the borderline high end as specified in the following ranges:

(1) 142 mg/dL≦TG≦420 mg/dL;
(2) 190 mg/dL≦TC≦252 mg/dL;
(3) HDL-C < 42 mg/dL for male or < 53 mg/dL for female; and
(4) 123 mg/dL≦LDL-C≦168 mg/dL.

Exclusion criteria included taking medications or supplements for lowering blood lipids, being allergic to adlay, and being pregnant or planning pregnancy. Volunteers were given a detailed explanation of the purpose, content, and protocol of the study as well as potential side effects. All subjects provided written informed consent and were recompensed for their participation in the study. They were also free to resign from the study at any time. The protocol was approved by the Research and Ethics Committee of Behavior and Social Sciences of National Taiwan University (approval ID: 2017HM020 and date: 20171221) and conducted in accordance with institutional and national guidelines and to the Declaration of Helsinki (revised 2013).

### 2.6. Sample Size Determination

**Figure 1:**
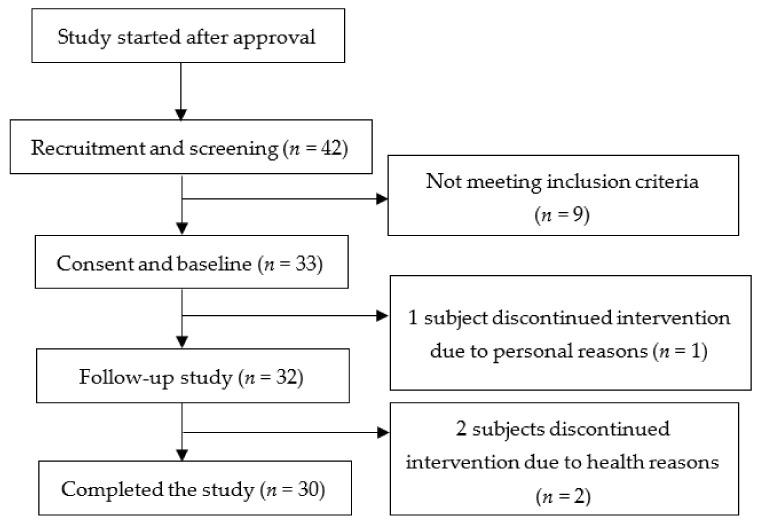
Flow diagram from recruitment to study completion.

The sample size was calculated using G*power 3.1.9.2 software. We determined the sample size by setting α probability to 5%, the power to 90%, and the effect size to 0.91 (a decrease in mean cholesterol levels by 10% between two groups (before-after) and variance at 11% based on previous literature [15]), and the number of subjects needed to achieve sufficient power was 15. More subjects were recruited to account for potential dropouts or losses to follow-up. Forty-two potential subjects were assessed for eligibility. Thirty-three subjects met the inclusion criteria after a blood lipid assessment was performed in our contracted health center and started the study. One participant resigned for personal reasons, and two others were asked to resign for taking hypolipidemic drugs during the experimental period. A total of 30 subjects completed the study (Figure 1).

### 2.7. Dietary Interventions

The test sample was formulated to consist of 90% by weight of the EDA powder and 10% by weight of fibersol^®^-2. Subjects were provided the test sample packed in sachets of 40 g for females and 55 g for males in order to provide the recommended daily calorie intake for breakfast staple according to the Health Promotion Administration, Ministry of Health and Welfare, Taiwan. The EDA powder was to be consumed at breakfast along with low-fat milk or unsweetened soymilk as the sole carbohydrate source and replacing other starchy and sugary foods, such as bread, fruits, and any foods with added sugar normally consumed at breakfast. Subjects were given sufficient sachets along with instructions for consuming the powder when the subjects came to the clinic for baseline and follow-up examinations every four weeks. The experimental duration lasted 12 weeks, and all subjects were asked not to modify their regular lifestyle and dietary habits during this period. Blood samples were drawn, and height, body weight, waist circumference, and blood pressure were recorded at baseline and at each follow-up every four weeks until the end of the intervention. To monitor changes in dietary intakes that play an important role in regulating blood lipid levels, 24-h dietary recalls were collected from participants at baseline and at each follow-up for a total of four times. No adverse events were reported by the subjects.

### 2.8. Serum Lipids Assays

Blood samples were drawn in the morning after an overnight fast. The serum levels of TG, TC, LDL-C, and HDL-C were analyzed using commercially available kits according to the manufacturer’s instructions in conjunction with UniCel^®^ DxC 800 and Synchron^®^ Systems Multi Calibrator (Beckman Coulter, Inc., Brea, CA, USA).

### 2.9. Outcome Measures

Participants in this study had abnormal levels of one or more blood lipids (single or mixed dyslipidemia). The primary outcome was changes in any of the four blood lipids measured (TC, TG, LDL-C, or HDL-C). Changes in blood lipid levels in the three follow-ups at weeks 4, 8, and 12 were compared to baseline values. Self-reported side effects and acceptability to the taste and texture of EDA were also recorded.

### 2.10. Statistical Analysis

All data were expressed as mean ± standard deviation (SD). For the in vitro digestibility experiments, data were analyzed by Student’s *t*-test with statistical significance defined at *p* < 0.05 and computed with SAS 9.4 (SAS Institute Inc., Ellicott, MD, USA). For the dietary intervention study, subjects were analyzed based on their initial blood lipid status and classified into three groups: hypercholesterolemia group, high LDL group, and low HDL group; one subject may qualify for more than one group, and the number of subjects in each group was indicated. Comparisons between baseline and every 4-week measurement were made using repeated-measures one-way analysis of variance (ANOVA) followed by Dunnett’s multiple comparisons test with statistical significance defined at *p*<0.05.

## 3. Results

### 3.1. Properties of Extruded Dehulled Adlay (EDA) Powder

#### 3.1.1. In Vitro Digestibility of EDA Powder

**Figure 2:**
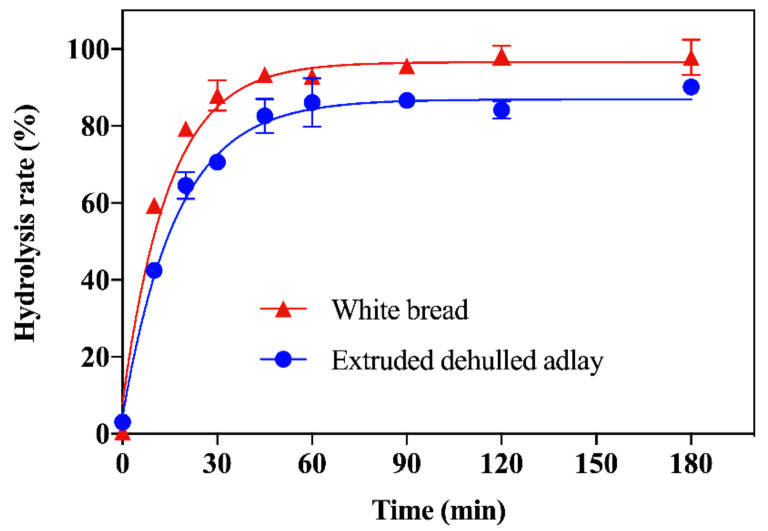
Starch hydrolysis curves for extruded dehulled adlay and white bread as reference. Data are expressed as mean ± SD (*n* = 3).

We processed dehulled adlay of cultivar TCS4 as described in the Materials and Methods. The in vitro digestion rate of the EDA powder was measured using the Englyst method [11], which classifies starch into three fractions, namely RDS, SDS, and RS, based on their rate of breakdown into glucose during controlled enzymatic hydrolysis. The starch hydrolysis curves are shown in Figure 2, and the results of equilibrium concentration (C∞) and kinetic constant (k) of the EDA powder are shown in Table 1.

Compared to white bread, the hydrolysis rate of the EDA powder was significantly slower at 15, 60, and 120 min of the enzymatic reaction, and significantly lower equilibrium concentration and kinetic constant were observed, indicating an overall slower enzymatic breakdown. The starch composition is shown in Table 2. The EDA powder had 15% lower RDS and 36% higher SDS as well as a 7.8-fold increase in RS compared to white bread. In addition, SDS and RS summed to comprise about 32% of the starches in the EDA powder.

#### 3.1.2. Phytosterols and Phenolic Compounds in EDA Powder

Phytosterols and phenolics are two of the most prominent bioactive compounds reported in adlay. The contents of the phytosterols campesterol, stigmasterol, and β-sitosterol as well as the phenolic compounds in the EDA powder are shown in Table 3. The three phytosterols summed up to 75.63 mg/g powder. The most abundant phenolic compounds in the EDA powder were di-coumaroylspermidine-1, di-coumaroylspermidine-2, chrysoeriol, and kaempferol, which amounted to 53.30 μg/g. Having determined the in vitro digestibility characteristics and the contents of phenolics and phytosterols, we prepared the EDA powder into the test sample for the subsequent human dietary intervention study.

### 3.2. Human Dietary Intervention

A total of 30 subjects (men = 5, women = 25), with a mean age of 52.77 ± 12.32 years, completed the study. Subjects were classified into three groups based on their initial blood lipid status (single dyslipidemia) determined during recruitment and screening: hypercholesterolemia group (*n* =26) for subjects having total cholesterol levels between 190 and 252 mg/dL, high LDL group (*n* = 22) for subjects having low-density lipoprotein cholesterol levels between 123 and 168 mg/dL, and low HDL group (*n* = 11) for subjects having high-density lipoprotein cholesterol levels below 42 mg/dL for males and 53 mg/dL for females. The number of subjects who had hypertriglyceridemia, defined as having triglyceride levels between 142 and 420 mg/dL, was not sufficient (*n* = 7), and thus, they were not analyzed separately. The baseline characteristics of all subjects (*n* = 30) and of each dyslipidemia group are shown in Table 4. Generally, the subjects did not identify any side effect associated with the ingestion of the test product and reported favorable tolerance regarding its taste and texture.

#### 3.2.1. The Effects of EDA on Subjects in the Hypercholesterolemia Group

After 12 weeks of consuming the EDA powder, there was a significant decrease in TC from 230.27 ± 17.78 mg/dL to 220.42 ± 22.88 mg/dL and HDL-C from 55.54 ± 14.19 mg/dL to 52.81 ± 12.42 mg/dL (*p*<0.05) in the hypercholesterolemic subjects (Table 5). No significant change in TG and LDL-C was observed, and the TC to HDL-C ratio (TC/HDL-C) showed no significant difference after the intervention. We then analyzed the population 50 years and above in this group and found a significant reduction in serum TG levels at Week 12 (Table 6).

#### 3.2.2. The Effects of EDA on Subjects in the High LDL Group

After the 12-week intervention, a significant reduction was seen in serum HDL-C levels, but the TC/HDL-C ratio was not significantly changed (Table 7). We narrowed the age range to 50 years and above and observed a significant reduction in serum TC levels in addition to a decrease in HDL-C (Table 8). However, the ratio of TC/HDL-C in this age range was not significantly altered after the intervention.

#### 3.2.3. The Effects of EDA on Subjects in the Low HDL Group

At the end of the intervention period, serum TG, TC, LDL-C, and HDL-C levels remained unchanged after statistical analysis although a transient increase in HDL-C was observed by week 8, resulting in a significant decrease in TC/HDL-C ratio (*p* = 0.037), but it became non-significant by week 12 (Table 9).

## 4. Discussion

Previous studies show that the rate of carbohydrate digestion of starchy foods can be modified by the source, processing, and culinary preparation [16]. Compared to the reference sample, the EDA powder, after extrusion and milling, had a lower in vitro digestibility. We attributed the lower digestion rate to its lower content of RDS and higher content of SDS and RS, which prolonged the enzymatic conversion to glucose. Furthermore, SDS and RS combined made up about 32% of total starch in the EDA powder. A previous study reported that consuming breakfast cereal containing 28% SDS+RS resulted in significantly lower postprandial glucose and insulin release, showing potential for improving metabolic syndrome [17].

Furthermore, a review of similar studies concluded that higher SDS are beneficial for the management of hyperlipidemia [18]. Since the population with mild to moderate dyslipidemia, which is at risk of developing CVDs, is larger than the population already diagnosed with CVDs and may be less careful about cardiovascular health, we recruited subjects from this population (criteria specified in Materials and Methods) and investigated the impact of consuming the EDA on their blood lipid levels. This was an open-label, non-controlled study using pre- and post-intervention comparisons of the same set of participants. In general, no statistically significant differences were found in weight, waist circumference, and blood pressure after the 12-week intervention. In addition, macronutrient analysis of the dietary records from the participants also showed no significant difference between baseline and at each follow-up (Tables S1–S4), minimizing the effect background diet may have on blood lipid variations detected in this study. With regards to blood lipid levels, a reduction in TC levels after 12 weeks of EDA consumption was observed in the hypercholesterolemia group. We also observed that subjects in the high LDL group exhibited reduced mean TC values from 229.27 ± 15.91 to 220.05 ± 21.72 mg/mL (Table 7). However, there were also significant reductions in HDL-C levels at weeks 4 and 12 in both groups. Nonetheless, the TC/HDL-C ratio, which is an indicator of ischemic heart disease, was not altered significantly in either group, suggesting the decrease in HDL-C level was unlikely to be associated with increased cardiovascular risk. Elevations in TC/HDL-C as well as non-HDL cholesterol levels, on the other hand, are better indicators of cardiovascular risk [19]. Meanwhile, there was an increase in mean HDL-C values in the low HDL group at week 8 compared to baseline, which resulted in a significant decrease in the TC/HDL-C ratio (Table 9). Nevertheless, the HDL-C level decreased later at week 12, and the TC/HDL-C ratio was not different from baseline. A general observation on the values of the blood lipids showed a U-shaped trend, where TG, TC, and LDL-C decreased after four weeks of EDA consumption but increased from Week 4 to 8 and then decreased again from week 8 to 12, whereas HDL-C had an opposite trend. This might suggest long-term consumption of EDA is required for seeing consistent blood lipid changes.

Aging is associated with lipid imbalance and increases in adiposity [20]; therefore, we decided to stratify subjects and analyze those over 50 years old (middle aged and beyond). The effect of age was evident in the hypercholesterolemia group, where there was a significant decrease in both TC and TG levels (Table 6) though the TC/HDL-C ratios remained not significantly changed despite decreases in HDL-C levels. There was also a significant decrease in TC levels at weeks 4 and 12 in the high LDL subjects above 50 years old (Table 8). We propose this enhanced effect of EDA in the middle-aged probably comes from the fact that advancing age increases the risk of insulin resistance due to skeletal muscle aging, which is associated with increased oxidative stress and inflammation and promotes hepatic lipogenesis [21]. Insulin resistance not only induces chronic hyperglycemia but also alters lipid metabolism [22]. Studies suggest that SDS may lower plasma lipids by prolonging intestinal lipid absorption and providing a sustained release of glucose, which insults in a lower insulin response to food and less lipid storage [23,24]. In addition, RS, also known as soluble dietary fiber, may lower plasma cholesterol by delaying gastric emptying, interfering with intestinal absorption, and increasing its excretion [25]. Consistently, the higher contents of SDS and RS of the EDA offers a favorable dietary formula to lower insulin-reduced lipidemic response following food ingestion, alleviating insulin resistance and thereby reducing TC and TG in the long term.

The decrease in blood lipid levels in the subjects may also come from the collectively greater intake of phytosterols and phenolic compounds in the EDA (Table 3). By calculation, consumption of 40 g of the EDA product provides 2.7 g phytosterols per day, which meets the recommended intake of dietary phytosterols that provides cardioprotective effects [26]. In addition, Li et al. [27] reported a positive association of higher dietary phytosterols with reductions in serum TC and LDL-C as well as the prevalence of obesity. Although the reduction in serum LDL-C was not statistically significant in our subjects despite a hypocholesterolemic effect, we did observe a reduction in mean LDL values. Several phenolic compounds have also shown hypolipidemic potential. For example, ρ-coumaric acid has been shown to decrease both plasma and tissue levels of TC and TG in diabetic rats [28]. Chrysoeriol, a flavone that is also present in citrus peels and rooibos, has been reported to protect against lipid oxidation and exhibit anti-inflammatory and anti-cardiotoxic actions in vitro [29] and anti-obesity effects by reducing hepatic cholesterol content in high-fat-diet-induced mice [30]. Kaempferol is also found to alleviate hyperlipidemia by reducing visceral fat deposits and plasma lipid levels in high-fat-diet-fed obese rats [31]. Similarly, hesperetin shows cholesterol-lowering and anti-oxidative functions in hypercholesterolemic hamsters. Furthermore, in hyperlipidemic subjects, ferulic acid supplementation for six weeks improves the lipid profile, oxidative stress, and inflammatory status [32]. Another component in our dehulled adlay formulation, fibersol^®^-2, is composed of 90% resistant maltodextrin (RMD) and is also a dietary fiber. A recent randomized, placebo-controlled study by Kitagawa and colleagues involving 140 healthy subjects revealed that consumption of 5 g RMD three times daily for three months did not cause reductions in blood TC, TG, or LDL-C though an increase in HDL-C and a decrease in visceral adipose tissue were observed [33]. The addition of 10% *w/w* fibersol^®^-2 in our formulation provided a daily dose of 3.6 g or 4.95 g RMD per 40-g or 55-g test sample, respectively. We proposed that this low dose would not be bioactive and that the lipid-lowering effect seen in our subjects was contributed mainly by the EDA.

This preliminary dietary intervention study followed a single-arm design, and placebo effect was not controlled for and may have played a role in the outcomes measured. The power and effect size did not meet predefined values; the results may thus not be statistically sufficient to confirm a significant finding, especially the low HDL group due to smaller sample size. However, the reductions of TC levels in high LDL subjects above 50 years old and of both TC and TG levels in hypercholesterolemic subjects above 50 years old, accompanied by the absence of reported adverse events and general tolerability of the test product, may warrant further investigation into the efficacy of the EDA in the middle-age population in alleviating dyslipidemia. Suggestions for a future randomized, controlled trial include involving more subjects to increase study power and including a comparator group to eliminate placebo effect and measures to assess and reinforce protocol compliance to resolve the U-shaped trend in the blood lipid changes.

## 5. Conclusions

In summary, the EDA contains about 32% SDS and RS combined. Consuming the EDA powder modulated blood lipid levels in dyslipidemic subjects, especially TG and TC levels in subjects above 50 years old with elevated serum TC, possibly contributed by the combination of SDS, RS, and phytochemicals present in the EDA, including phytosterols and phenolic compounds, which have been reported to exhibit lipid-lowering effects. In conclusion, EDA displays a potential for controlling blood lipids, and the effect is more distinct in the middle-aged population. Overall, the consumption of EDA was safe and well tolerated and warrants further investigations into its efficacy in future randomized, controlled trials.

## Figures and Tables

**Table 1:** The starch hydrolysis parameters of extruded dehulled adlay powder and white bread as reference sample.

Sample	C_∞_ (%)	k
White bread	96.98 ± 2.45	0.088 ± 0.006
Extruded dehulled adlay	89.08 ± 4.2 *	0.070 ± 0.002 **
*p* **Value**	0.048	0.008

Data are expressed as mean ± SD (*n* = 3). Asterisks indicate significant difference by Student’s *t*-test between the samples (* *p* < 0.05, ** *p* < 0.01). C_∞_, concentration at equilibrium at the end of reaction; k, the kinetic constant.

**Table 2:** The contents of rapidly digestible starch (RDS), slowly digestible starch (SDS), and resistant starch (RS) of extruded dehulled adlay powder and white bread.

Sample	RDS (%)	SDS (%)	RS (%)
White bread	80.35 ± 1.59	16.63 ± 2.30	1.20 ± 1.58
Extruded dehulled adlay	68.42 ± 2.25 **	22.61 ± 1.94 *	9.36 ± 4.20 *
*p* **Value**	0.002	0.026	0.035

Data are expressed as mean ± SD (n = 3). Asterisks indicate significant difference by Student’s *t*-test between the samples (* *p* < 0.05, ** *p* < 0.01).

**Table 3:** The contents of phytosterols and phenolic compounds (in quercetin equivalent, QE) in extruded dehulled adlay powder.

Phytosterols	Content (mg/g)	Standard Curves	Correlation Coefficients	LOQ (μg/mL)
Campesterol	9.76 ± 0.52	y = 1.17432 + 0.00212571	0.99989	0.1
β-Sitosterol	56.2 ± 1.31	y = 1.15789x + 0.00194746	0.99993	
Stigmasterol	9.67 ± 0.20	y = 1.117x + 0.00224211	0.99987	
**Phenolic compounds**	**Content in QE (μg/g)**	**Standard curve (quercetin)**	**Correlation coefficient**	**LOQ (μg/mL)**
Chrysoeriol	0.98 ± 0.34	y = 26242392x − 1554452	0.99982	0.04
Coumaric acid isomer-1	0.34 ± 0.01			
Coumaric acid isomer-2	0.21 ± 0.05			
*ρ*-Coumaric acid	0.20 ± 0.05			
di-Coumaroylspermidine-1	8.87 ± 0.25			
di-Coumaroylspermidine-2	42.90 ± 0.16			
Eriodictyol	0.20 ± 0.05			
Ferulic aicd	0.35 ± 0.04			
Hesperetin	0.47 ± 0.14			
Kaempferol	0.55 ± 0.18			

**Table 4:** Baseline characteristics of all subjects and of each dyslipidemia group in the intervention study.

Characteristic	All Subjects (*n* = 30)	Hypercholesterolemia (*n* = 26)	High LDL (*n* = 22)	Low HDL (*n* = 11)
Age (y)	52.77 ± 12.32	54.69 ± 11.60	54.27 ± 12.56	48.00 ± 12.31
Male/Female ratio	5/25	5/21	4/18	0/11
Anthropometric values (cm)				
Height	160.85 ± 7.29	160.70 ± 7.82	160.72 ± 7.70	159.09 ± 5.92
Weight	65.02 ± 11.80	64.98 ± 12.40	65.19 ± 12.64	61.00 ± 8.62
Waist circumference	85.65 ± 9.38	85.73 ± 9.72	85.77 ± 10.31	82.18 ± 6.01
Body mass index (kg/m^2^)	25.03 ± 3.54	25.06 ± 3.68	25.14 ± 3.88	24.07 ± 2.95
Fasting serum lipids (mg/dL)				
Total cholesterol	221.80 ± 28.92	230.27 ± 17.78	229.27 ± 15.91	204.09 ± 35.48
HDL-C	54.17 ± 13.77	55.54 ± 14.19	57.23 ± 14.66	44.45 ± 4.03
LDL-C	143.17 ± 24.15	149.85 ± 16.01	150.23 ± 9.49	131.64 ± 33.76
Triglycerides	127.53 ± 81.51	130.69 ± 83.11	109.23 ± 52.75	143.18 ± 101.75
Blood pressure (mmHg)				
Systolic	123.33 ± 16.68	125.38 ± 16.06	127.27 ± 16.38	112.73 ± 12.72
Diastolic	78.53 ± 10.81	79.46 ± 10.49	80.27 ± 11.03	73.64 ± 9.24

Data are expressed as mean ± SD. LDL-C, low-density lipoprotein cholesterol; HDL-C, high-density lipoprotein cholesterol.

**Table 5:** Analysis of serum lipid profile in hypercholesterolemia group (*n* = 26).

	TG (mg/dL)		TC (mg/dL)		LDL-C (mg/dL)		HDL-C (mg/dL)		TC/HDL-C	
	Mean ± SD	*p* Value ^1^	Mean ± SD	*p* Value ^1^	Mean ± SD	*p* Value ^1^	Mean ± SD	*p* Value ^1^	Mean ± SD	*p* Value ^1^
Baseline	130.69 ± 83.11	-	230.27 ± 17.78	-	149.85 ± 16.01	-	55.54 ± 14.19	-	4.36 ± 0.95	-
Week 4	115.31 ± 56.83	0.152	223.88 ± 21.92	0.101	147.69 ± 17.77	0.801	52.92 ± 13.39 **	**0.009**	4.43 ± 0.91	0.759
Week 8	117.85 ± 64.07	0.571	229.50 ± 22.04	0.994	149.81 ± 18.36	>0.999	54.54 ± 14.55	0.768	4.48 ± 1.22	0.834
Week 12	114.77 ± 67.87	0.099	220.42 ± 22.88 *	**0.033**	144.35 ± 17.97	0.212	52.81 ± 12.42 *	**0.043**	4.34 ± 0.87	0.994

Data are expressed as mean ± SD. ^1^ *p* value: Significant difference (bolded number) versus baseline by repeated-measures one-way ANOVA followed by Dunnett’s multiple comparisons test (* *p* < 0.05, ** *p* < 0.01). TG, triglycerides; TC, total cholesterol; LDL-C, low-density lipoprotein cholesterol; HDL-C, high-density lipoprotein cholesterol.

**Table 6:** Analysis of serum lipid profile in hypercholesterolemic subjects above 50 years old (*n* = 20).

	TG (mg/dL)		TC (mg/dL)		LDL-C (mg/dL)		HDL-C (mg/dL)		TC/HDL-C	
	Mean ± SD	*p* Value ^1^	Mean ± SD	*p* Value ^1^	Mean ± SD	*p* Value ^1^	Mean ± SD	*p* Value ^1^	Mean ± SD	*p* Value ^1^
Baseline	131.50 ± 84.03	-	232.21 ± 18.99	-	149.25 ± 17.61	-	57.45 ± 16.64	-	4.22 ± 0.86	-
Week 4	111.70 ± 57.93	0.060	214.47 ± 21.64 *	**0.016**	147.25 ± 16.78	0.786	54.35 ± 14.10 **	**0.002**	4.32 ± 0.88	0.454
Week 8	105.85 ± 47.94	0.178	231.16 ± 21.92	0.991	152.00 ± 19.56	0.833	56.40 ± 15.05	0.850	4.38 ± 1.26	0.821
Week 12	106.80 ± 56.53 *	**0.020**	216.63 ± 23.69 **	**0.002**	141.30 ± 19.38	0.120	54.40 ± 12.78 *	**0.044**	4.14 ± 0.75	0.809

Data are expressed as mean ± SD. ^1^ *p* value: Significant difference (bolded number) versus baseline by repeated-measures one-way ANOVA followed by Dunnett’s multiple comparisons test (* *p* < 0.05, ** *p* < 0.01). TG, triglycerides; TC, total cholesterol; LDL-C, low-density lipoprotein cholesterol; HDL-C, high-density lipoprotein cholesterol.

**Table 7:** Analysis of serum lipid profile in high LDL group (*n* = 22).

	TG (mg/dL)		TC (mg/dL)		LDL-C (mg/dL)		HDL-C (mg/dL)		TC/HDL-C	
	Mean ± SD	*p* Value ^1^	Mean ± SD	*p* Value ^1^	Mean ± SD	*p* Value ^1^	Mean ± SD	*p* Value ^1^	Mean ± SD	*p* Value ^1^
Baseline	109.23 ± 52.75	-	229.27 ± 15.91	-	150.23 ± 9.48	-	57.23 ± 14.66	-	4.22 ± 0.96	-
Week 4	102.64 ± 41.19	0.686	221.5 ± 21.04	0.071	146.41 ± 12.96	0.454	54.36 ± 14.07 *	**0.012**	4.27 ± 0.89	0.905
Week 8	111.18 ± 61.56	0.984	227.91 ± 22.72	0.976	150.27 ± 16.23	>0.999	55.36 ± 15.69	0.378	4.43 ± 1.32	0.617
Week 12	103.95 ± 57.45	0.655	220.05 ± 21.72	0.084	145.23 ± 12.92	0.318	53.68 ± 13.16 *	**0.017**	4.28 ± 0.92	0.893

Data are expressed as mean ± SD. ^1^ *p* value: Significant difference (bolded number) versus baseline by repeated-measures one-way ANOVA followed by Dunnett’s multiple comparisons test (* *p* < 0.05). TG, triglycerides; TC, total cholesterol; LDL-C, low-density lipoprotein cholesterol; HDL-C, high-density lipoprotein cholesterol.

**Table 8:** Analysis of serum lipid profile in high LDL subjects above 50 years old (*n* = 16).

	TG (mg/dL)		TC (mg/dL)		LDL-C (mg/dL)		HDL-C (mg/dL)		TC/HDL-C	
	Mean ± SD	*p* Value ^1^	Mean ± SD	*p* Value ^1^	Mean ± SD	*p* Value ^1^	Mean ± SD	*p* Value ^1^	Mean ± SD	*p* Value ^1^
Baseline	102.19 ± 33.73	-	230.31 ± 16.74	-	149.63 ± 9.58	-	60.25 ± 14.91	-	4.00 ± 0.80	-
Week 4	93.38 ± 31.35	0.371	220.81 ± 19.58 **	**0.004**	145.38 ± 7.90	0.187	56.69 ± 14.87 **	**0.003**	4.08 ± 0.81	0.661
Week 8	93.69 ± 31.78	0.442	229.88 ± 22.43	>0.999	153.19 ± 16.91	0.784	58.00 ± 16.48	0.460	4.29 ± 1.40	0.602
Week 12	89.94 ± 24.28	0.136	216.25 ± 21.76 *	**0.021**	141.75 ± 13. 32	0.179	56.00 ± 13.64 *	**0.013**	4.01 ± 0.76	0.999

Data are expressed as mean ± SD. ^1^ *p* value: Significant difference (bolded number) versus baseline by repeated-measures one-way ANOVA followed by Dunnett’s multiple comparisons test (* *p* < 0.05, ** *p* < 0.01). TG, triglycerides; TC, total cholesterol; LDL-C, low-density lipoprotein cholesterol; HDL-C, high-density lipoprotein cholesterol.

**Table 9:** Analysis of serum lipid profile in low HDL group (*n* = 11).

	TG (mg/dL)		TC (mg/dL)		LDL-C (mg/dL)		HDL-C (mg/dL)		TC/HDL-C	
	Mean ± SD	*p* Value ^1^	Mean ± SD	*p* Value ^1^	Mean ± SD	*p* Value ^1^	Mean ± SD	*p* Value ^1^	Mean ± SD	*p* Value ^1^
Baseline	143.18 ± 101.75	-	204.09 ± 35.48	-	131.64 ± 33.76	-	44.45 ± 4.03	-	4.63 ± 0.85	-
Week 4	155.00 ± 96.58	0.931	204.09 ± 35.00	>0.999	129.45 ± 36.73	0.970	44.91 ± 7.74	0.984	4.67 ± 0.99	0.992
Week 8	119.64 ± 64.27	0.965	206.55 ± 45.51	0.966	131.00 ± 43.04	>0.999	48.00 ± 7.82	0.117	4.36 ± 0.84 *	**0.037**
Week 12	144.82 ± 79.19	>0.999	200.73 ± 30.45	0.949	124.73 ± 30.23	0.479	46.91 ± 7.45	0.172	4.39 ± 0.89	0.350

Data are expressed as mean ± SD. ^1^ *p* value: Significant difference (bolded number) versus baseline by repeated-measures one-way ANOVA followed by Dunnett’s multiple comparisons test (* *p* < 0.05). TG, triglycerides; TC, total cholesterol; LDL-C, low-density lipoprotein cholesterol; HDL-C, high-density lipoprotein cholesterol.

## Data Availability

The data that support the findings of this study are included in this article and Supplementary Materials. Further enquiries can be directed to the corresponding author.

## Supplementary Materials

The following supporting information can be downloaded at: https://www.mdpi.com/article/10.3390/foods11040493/s1, Figure S1: Standard curve for the quantitation of campesterol; Figure S2: Standard curve for the quantitation of stigmasterol; Figure S3: Standard curve for the quantitation of β-sitosterol; Table S1: Analysis of macronutrients from 24-h dietary recalls in all subjects (*n* = 30); Table S2: Analysis of macronutrients from 24-h dietary recalls in the hypercholesterolemia group (*n* = 26); Table S3: Analysis of macronutrients from 24-h dietary recalls in the high LDL group (*n* = 22); and Table S4: Analysis of macronutrients from 24-h dietary recalls in the low HDL group (*n* = 11).
