# Supplementary material for: Investigating the Impact of Extruded Dehulled Adlay with Specific In Vitro Digestion Properties on Blood Lipids in Subjects with Mild to Moderate Dyslipidemia
Source: Foods. 2022 Feb 9;11(4):493. doi: 10.3390/foods11040493 (PMC8871124; doi:10.3390/foods11040493)
Supplement: Supplementary file 1 [file foods-11-00493-s001.zip › Suppl Tables S1-4.pdf]

Table S1. Analysis of macronutrients from 24-h dietary recalls in all subjects (n = 30).

	Carbohydrates (g)		Fats (g)		Proteins (g)	
	**Mean ± SD**	***p* Value[1]**	**Mean ± SD**	***p* Value[1]**	**Mean ± SD**	***p* Value[1]**
Baseline	185.28 ± 37.86	-	64.21 ± 11.43	-	59.76 ± 11.02	-
Week 4	191.30 ± 36.89	0.841	63.67 ± 10.70	0.995	57.27 ± 10.90	0.689
Week 8	183.48 ± 38.36	0.994	62.11 ± 10.43	0.785	55.52 ± 11.07	0.286
Week 12	182.18 ± 23.54	0.973	62.26 ± 9.94	0.819	56.6 ± 9.34	0.520

Data were expressed as mean ± SD

[1]*p* value: Statistical analysis was performed to test difference between each follow-up versus baseline by repeated-measures one-way ANOVA

Table S2. Analysis of macronutrients from 24-h dietary recalls in the hypercholesterolemia group (n = 26).

	Carbohydrates (g)		Fats (g)		Proteins (g)	
	**Mean ± SD**	***p* Value[1]**	**Mean ± SD**	***p* Value[1]**	**Mean ± SD**	***p* Value[1]**
Baseline	188.13 ± 38.11	-	65.19±10.92	-	60.69±10.96	-
Week 4	190.7 ± 35.88	0.987	63.26±11.42	>0.999	57.90±11.53	0.681
Week 8	184.78 ± 38.72	0.973	62.19±10.59	>0.999	57.12±11.20	0.503
Week 12	181.67 ± 25.16	0.844	62.78±10.24	>0.999	57.14±9.90	0.507

Data were expressed as mean ± SD

[1]p value: Statistical analysis was performed to test difference between each follow-up versus baseline by repeated-measures one-way ANOVA

Table S3. Analysis of macronutrients from 24-h dietary recalls in the high LDL group (*n* = 22).

	Carbohydrates (g)		Fats (g)		Proteins (g)	
	**Mean ± SD**	***p* Value[1]**	**Mean ± SD**	***p* Value[1]**	**Mean ± SD**	***p* Value[1]**
Baseline	189.15 ± 36.86	-	64.49 ± 11.64	-	62.17 ± 10.93	-
Week 4	192.85 ± 38.38	0.975	63.67 ± 12.70	0.991	58.62 ± 11.36	0.563
Week 8	188.39 ± 41.25	>0.999	62.79 ± 12.16	0.929	57.85 ± 11.81	0.408
Week 12	178.7 ± 27.79	0.660	63.03 ± 9.17	0.953	56.25 ± 8.80	0.175

Data were expressed as mean ± SD

[1]*p* value: Statistical analysis was performed to test difference between each follow-up versus baseline by repeated-measures one-way ANOVA

Table S4. Analysis of macronutrients from 24-h dietary recalls in the low HDL group (*n* = 11).

	Carbohydrates (g)		Fats (g)		Proteins (g)	
	**Mean ± SD**	***p* Value[1]**	**Mean ± SD**	***p* Value[1]**	**Mean ± SD**	***p* Value[1]**
Baseline	167.42 ± 39.69	-	60.62 ± 10.01	-	54.52 ± 9.22	-
Week 4	177.88 ± 35.57	0.803	65.17 ± 4.74	0.394	55.00 ± 5.65	0.998
Week 8	166.68 ± 29.04	>0.999	60.41 ± 9.29	>0.999	55.96 ± 9.84	0.953
Week 12	173.36 ± 26.99	0.954	60.4 ± 6.07	>0.999	55.23 ± 6.86	0.994

Data were expressed as mean ± SD

[1]*p* value: Statistical analysis was performed to test difference between each follow-up versus baseline by repeated-measures one-way ANOVA
